# Outcomes after helicopter versus ground emergency medical services for major trauma--propensity score and instrumental variable analyses: a retrospective nationwide cohort study

**DOI:** 10.1186/s13049-016-0335-z

**Published:** 2016-11-29

**Authors:** Asuka Tsuchiya, Yusuke Tsutsumi, Hideo Yasunaga

**Affiliations:** 1 Department of Clinical Epidemiology & Health Economics, School of Public Health, Graduate School of Medicine, The University of Tokyo, 7-3-1, Hongo, Bunkyo-ku, Tokyo, 1130033 Japan; 2 Department of Emergency and Critical Care Medicine, National Hospital Organization Mito Medical Center, 280, Sakuranosato, Ibarakimachi, Higahi-Ibarakigun, Ibaraki, 3113193 Japan

**Keywords:** Helicopter emergency medical service, Ground emergency medical service, Trauma, Propensity score, Instrumental variable, Mortality, Japan Trauma Data Bank

## Abstract

**Background:**

Because of a lack of randomized controlled trials and the methodological weakness of currently available observational studies, the benefits of helicopter emergency medical services (HEMS) over ground emergency medical services (GEMS) for major trauma patients remain uncertain. The aim of this retrospective nationwide cohort study was to compare the mortality of adults with serious traumatic injuries who were transported by HEMS and GEMS, and to analyze the effects of HEMS in various subpopulations.

**Methods:**

Using the Japan Trauma Data Bank, we evaluated all adult patients who had an injury severity score ≥ 16 transported by HEMS or GEMS during the daytime between 2004 and 2014. We compared in-hospital mortality between patients transported by HEMS and GEMS using propensity score matching, inverse probability of treatment weighting and instrumental variable analyses to adjust for measured and unmeasured confounding factors.

**Results:**

Eligible patients (*n* = 21,286) from 192 hospitals included 4128 transported by HEMS and 17,158 transported by GEMS. In the propensity score-matched model, there was a significant difference in the in-hospital mortality between HEMS and GEMS groups (22.2 vs. 24.5%, risk difference −2.3% [95% confidence interval, −4.2 to −0.5]; number needed to treat, 43 [95% confidence interval, 24 to 220]). The inverse probability of treatment weighting (20.8% vs. 23.9%; risk difference, −3.9% [95% confidence interval, −5.7 to −2.1]; number needed to treat, 26 [95% confidence interval, 17 to 48]) and instrumental variable analyses showed similar results (risk difference, −6.5% [95% confidence interval, −9.2 to −3.8]; number needed to treat, 15 [95% confidence interval, 11 to 27]). HEMS transport was significantly associated with lower in-hospital mortality after falls, compression injuries, severe chest injuries, extremity (including pelvic) injuries, and traumatic arrest on arrival to the emergency department.

**Conclusions:**

HEMS was associated with a significantly lower mortality than GEMS in adult patients with major traumatic injuries after adjusting for measured and unmeasured confounders.

**Electronic supplementary material:**

The online version of this article (doi:10.1186/s13049-016-0335-z) contains supplementary material, which is available to authorized users.

## Background

Helicopter emergency medical services (HEMS) have about a half-century history. Early adopters, such as Germany and the United States, have been operating emergency medical helicopters since 1970 [1], and HEMS have become an important component of pre-hospital care for trauma patients in many countries [2–5]. HEMS can provide faster transport of severely injured patients to highly specialized facilities than ground emergency medical services (GEMS).

Several recent, well-designed studies suggest that HEMS was associated with improved survival [6–11], however other studies reported no significant difference [12–14]. The differences in the findings were related to the great diversity of EMS between different countries, as well as different study designs and populations [15–17]. Additionally, all these studies were observational in design, and were not able to account for unmeasured confounders such as degree of emergency other than vital signs. Therefore, the benefits of HEMS remain controversial.

HEMS require substantially higher costs and more training of health care professionals than GEMS. Minor injury patients may not be appropriate candidates for HEMS, because there may be limited scope for an improvement in outcome for such patients and greater expense related to HEMS [18–20]. However, it remains uncertain which types of patients are likely to benefit from HEMS.

The aims of the present study were: (i) to compare mortality between HEMS and GEMS in severely injured patients while adjusting for measured and unmeasured confounders; and (ii) to analyze the effects of HEMS in various subpopulations.

## Methods

This study was approved by the institutional review board of National Hospital Organization Mito Medical Center, which waived the requirement for informed patient consent because of the anonymous nature of the data.

This study was retrospective nationwide cohort study. Data were obtained from the Japan Trauma Data Bank (JTDB) during the years 2004–2014. The JTDB is the largest repository of national trauma data in Japan. Data were collected from 244 participating hospitals (197 tertiary, 47 secondary-level emergency hospitals), and in 2014, about 71% (197/279) of tertiary-level emergency hospitals in Japan participated in the database and 93% (184,521/198,745) of data were collected from tertiary-level hospitals. Tertiary and secondary-level hospitals are authorized by the Ministry of Health, Labour and Welfare. Tertiary-level hospitals are equivalent role to level 1 trauma centers in Europe and the United States, and are capable of providing 24-h specialty care in areas such as general surgery, cardiovascular surgery, orthopedic surgery, neurosurgery, anesthesiology, emergency medicine, radiology, internal medicine and critical care [21]. The JTDB was started in 2003 by the Japanese Association for Trauma Surgery (Trauma Registry Committee) and the Japanese Association for Acute Medicine (Committee for Clinical Care Evaluation). The Association for Japan Trauma Care Research plays the leading role in the training of Abbreviated Injury Scale (AIS)-certified trauma registry coders. Data are prospectively and continuously recorded through a web-based format, and the data are compiled in a data server at the Association for Japan Trauma Care Research. The Association for Japan Trauma Care Research cleanses the data and publishes an annual report [22].

The database contains each patient’s demographic data (age, sex, and vital signs at the scene of injury and in the emergency department); the mechanism of injury; pre-existing medical conditions according to the International Classification of Diseases, 10th revision; diagnoses; surgical and interventional procedures; severity of injury; and patient disposition. Diagnosis of injury is recorded according to the AIS using AIS 90 Update 98, and patients with AIS ≥3 are recorded. The severity of anatomic injuries is evaluated using the injury severity score (ISS). Level of consciousness is evaluated using the Japan Coma Scale score, which is recorded for all patients on admission. This score correlates well with the Glasgow Coma Scale; a neurologic dysfunction score of 100 points on the Japan Coma Scale is equivalent to 6–9 on the Glasgow Coma Scale. Patients were categorized into four groups based on Japan Coma Scale score 0 (Grade 0, alert); 1–3 (Grade 1, delirium); 10–30 (Grade 2, somnolence); and 100–300 (Grade 3, coma) [23].

### HEMS

In Japan, HEMS was first introduced in 2000 and has spread to many regions. In 2014, there were 22,463 helicopter transports and the number is gradually increasing every year. The Japanese HEMS are similar to those in European countries and have a physician-based pre-hospital approach to emergency patients. One or two physicians and a nurse are transported to the scene by helicopter. Physicians are board-certified in fields such as acute care, surgery, anesthesiology or aeromedical services and have received advanced trauma life support training. Each helicopter covers a radius of about 50 km during daylight hours. HEMS are based at a tertiary-level emergency hospital and can be dispatched according to the information given during the emergency call from the fire department. HEMS dispatch can also be requested by the GEMS upon assessment of the patient at the scene. GEMS first rescues the injured patient, and then transports them to a location where HEMS can land safely (the “rendezvous point”). Then the HEMS team provides emergency care such as endotracheal intubation, chest tube drainage, emergency tracheotomy or thoracotomy with aortic clamping in the ambulance using various medications. This system is called the “rendezvous system”. After emergency care, HEMS transports the patient to the tertiary care hospital.

### GEMS

Japanese GEMS consist of emergency medical technicians or paramedics trained in advanced life support and pre-hospital trauma life support, and fire fighters trained in basic life support. GEMS are allowed to perform several procedures according to fixed protocols set by the Ministry of Health, Labour and Welfare, including venous cannulation, crystalloid infusion, early defibrillation, endotracheal intubation without muscle relaxants, and treatment with several medications for cardiopulmonary arrest [24].

### Inclusion criteria

A total of 198,744 patients were enrolled in the JTDB from 2004 to 2014. Inclusion criteria for the study were: (i) age 15 years or older; (ii) transport by HEMS or GEMS; (iii) direct transport from the scene of injury; (iv) hospital admission from January 2004 to December 2014; (iv) hospital arrival between 8:30 and 18:00 (daytime); and (v) ISS ≥16 points. We excluded (i) patients who had no vital signs (traumatic arrest) at the time of GEMS arrival; (ii) those with burns (because burns are different from other blunt or stab injuries in that they are not accompanied by bleeding or obstructive shock, which can cause death in minutes); and (iii) those who experienced falls from standing to flat ground (because such falls generally do not cause serious injuries). Patients with no outcome data or recorded sex were also excluded.

Patients were stratified into two groups according to transport by HEMS or GEMS. The outcomes of interest were in-hospital mortality. Subgroup analyses were performed to compare mortalities according to cause of injury, injury distribution, and hospital type. We also analyzed: (i) initial vital signs when patients arrived to the emergency department; (ii) the proportion of patients who were in traumatic arrest on arrival to the emergency department; (iii) the mortality rate of patients who were not in traumatic arrest on arrival to the emergency department; and (iv) the interval between fire department dispatch and emergency department arrival, and the interval between emergency department arrival and blood transfusion or definitive care such as emergency surgery or transarterial embolization.

### Statistical analyses

Quantitative variables were grouped based on Trauma and Injury Severity Score methods, which are commonly used in medical practice [25]. Covariates were carefully selected based on the assumption that they were not directly affected by the intervention. The Wilcoxon rank-sum test was used to compare (i) the time from dispatch to emergency department arrival, (ii) the time from emergency department arrival to blood transfusion, and (iii) the time from emergency department arrival to definitive care.

### Propensity score analyses

One-to-one propensity-score matching was performed between the HEMS and GEMS groups [26–28]. To estimate the propensity score, a logistic regression model was used with the following independent variables: age, sex, preexisting medical conditions, mechanism of injury, ISS, injury distribution with AIS ≥ 3, pre-hospital vital signs (systolic blood pressure, respiratory rate, heart rate and Japan Coma Scale scores), and the accident date. Vital sign components were categorized, and missing data were included as a missing category. The C-statistic for evaluating the goodness of fit was calculated. Using a nearest-neighbor matching method, each patient in the HEMS group was matched with a patient in the GEMS group without replacement, with the closest estimated propensity within a caliper (≤0.2 of the pooled standard deviation of propensity scores). We examined the balance in the baseline variables between the propensity-matched HEMS and GEMS groups using standardized differences, where >10% was regarded as imbalanced [29, 30]. We also used a propensity score method for inverse probability of treatment weighting (IPTW) using the same population as that in the instrumental variable analysis (mentioned below). Each patient was weighted by the inverse probability of being in the observed group [31, 32].

In the propensity score analyses, we calculated the risk differences and their 95% confidence intervals (CI) and the number needed to treat in in-hospital mortality. In-hospital mortality was compared between the HEMS and GEMS groups according to subpopulations of (i) cause of injury, (ii) injury distribution (AIS ≥3), and (iii) type of hospital (tertiary or secondary), using chi-square tests.

In addition, initial vital signs at the emergency department, including systolic blood pressure, heart rate, respiratory rate, Japan Coma Scale, Glasgow Coma Scale, and body temperature, were compared between the HEMS and GEMS groups using the standardized difference.

### Instrumental variable analysis

Propensity score analysis cannot remove hidden biases caused by unmeasured confounders. We therefore conducted an instrumental variable (IV) analysis as a confirmatory analysis of our propensity score analyses. The key assumptions of an IV analysis are that the IV is highly correlated with the treatment, but is otherwise not correlated with any unmeasured variables, so that it does not affect patient outcomes except through treatment [33, 34].

In a pre-hospital setting, wide variations in the frequency of HEMS use for major trauma may be related to the local fire department’s policies and preferences. Some fire departments prefer activating HEMS at same time as dispatching the emergency call, and others activate HEMS after GEMS assessment. Such preferences inevitably differ between regions based on demographic, geographic, and health care resource considerations. We first classified each patient by transportation mode, and then examined the most recent prior transportation mode used by the same fire department for any other patient in the cohort. We used “last transportation mode” as an instrumental variable. In this approach, if the last transportation mode used by the same fire department was HEMS, the fire department was regarded as an HEMS user for that patient. Otherwise, the fire department was regarded as a GEMS user [35, 36]. The last mode of transport rule was used as a surrogate for actual treatment, and was considered independent of patient characteristics and not directly related to outcome [35]. In the IV analysis, we excluded patients in some regions where HEMS was not available. We also excluded patients during the periods before HEMS was introduced in each region, patients with no associated fire department data and fire departments that had fewer than 10 patient transports [37]. We used a two-stage least-squares regression with the covariates (age, sex, preexisting medical conditions, cause of injury, ISS, pre-hospital systolic blood pressure, pre-hospital respiratory rate, pre-hospital heart rate, pre-hospital Japan Coma Scale scores, and the accident date) for IV analysis to estimate the risk difference and its 95% CI for in-hospital mortality using Stata/MP 14.0 (Stata Corp., College Station, TX, USA). We confirmed the validity of the instrument by testing its association with our main predictor of the actual transportation mode using partial F-statistics. Partial F-statistics > 10 were regarded as valid instruments [38]. To further assess the validity of our instrument, we examined the covariate balance [39].

The threshold for significance was a *P*-value < 0.05. For the subgroup analyses, we used the Bonferroni correction to counteract the problem of multiple comparisons [40]. With the Bonferroni correction, rejecting null hypotheses at *p* < α/*m* controls the familywise error rate, where α = 0.05 and *m* denotes the total number of null hypotheses. Because we performed 24 subgroup analyses, the significance level for their *P*-values was set as < 0.002. All statistical analyses were conducted using IBM SPSS version 22 (IBM Corp., Armonk, NY, USA) and Stata/MP 14.0.

## Results

**Fig. 1:**
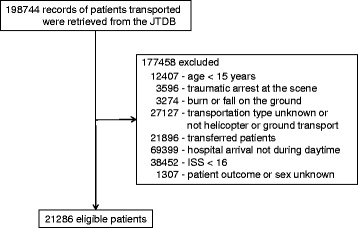
Study Flow Diagram Detailing the Stratification and Selection of Patients in the JTDB (2004–2014). JTDB indicates Japan Trauma Data Bank; ISS, injury severity score; HEMS, helicopter emergency medical services; GEMS, ground emergency medical service

A total of 21,286 eligible patients with trauma were treated at 192 hospitals during the study period (Fig. 1). There were 4128 HEMS patients (3143 male, 985 female; mean age: 58.1 years; range, 84 years) and 17,158 GEMS patients (11,906 male, 5252 female; mean age: 57.3 years; range, 86 years), from which 3980 propensity score-matched pairs were generated. The C-statistic was 0.70 (95% CI 0.69 to 0.71) in the model for calculating propensity scores.

Table 1 shows the baseline characteristics of the unmatched and propensity score-matched groups. When the unmatched groups were compared, patients were more likely to be transported by HEMS if they were injured in an automobile crash or by severe compression. Patients transported by HEMS had higher ISS and a higher proportion of chest, abdominal, spinal, and extremity (including pelvic) injuries than those transported by GEMS. The variables of the propensity score-matched groups were well balanced.

**Table 1:** Baseline patients characteristics in the unmatched and propensity score-matched groups

	Unmatched groups					Matched groups				
	HEMS		GEMS		SD, %	HEMS		GEMS		SD, %
	*n* = 4128	(%)	*n* = 17158	(%)		*n* = 3980	(%)	*n* = 3980	(%)	
Age, years										
15 - 55	1586	(38.4)	6923	(40.3)	2.5	1536	(38.6)	1516	(38.1)	-2.3
56 - 64	708	(17.2)	2740	(16.0)	4.3	681	(17.1)	697	(17.5)	4.1
≥ 65	1834	(44.4)	7495	(43.7)	-5.8	1763	(44.3)	1767	(44.4)	-0.8
Sex male	3143	(76.1)	11906	(69.4)	16.4	3016	(75.8)	3039	(76.4)	-1.2
Systolic blood pressure, mm Hg^a^										
< 90	318	(7.7)	1120	(6.5)	5.9	309	(7.8)	315	(7.9)	2.7
90 to 109	444	(10.8)	2078	(12.1)	-2.6	436	(11.0)	456	(11.5)	1.0
≥ 110	2146	(52.0)	10614	(61.9)	-23.9	2107	(52.9)	2105	(52.9)	-1.2
missing	1220	(29.6)	3346	(19.5)	26.0	1128	(28.3)	1104	(27.7)	-0.9
Heart rate, beats/min^a^										
< 60	228	(5.5)	817	(4.8)	3.6	221	(5.6)	241	(6.1)	-2.9
60 to 99	2048	(49.6)	10144	(59.1)	-21.8	2015	(50.6)	2109	(53.0)	1.2
≥ 100	851	(20.6)	3862	(22.5)	-2.7	835	(21.0)	746	(18.7)	1.2
missing	1001	(24.2)	2335	(13.6)	29.0	909	(22.8)	884	(22.2)	-1.0
Respiratory rate, breaths/min^a^										
< 10 or > 29	781	(18.9)	3030	(15.0)	10.2	770	(19.3)	743	(18.7)	0.8
10 to 29	1818	(44.0)	13175	(65.3)	-42.5	1805	(45.4)	1822	(45.8)	-0.6
missing	1529	(37.0)	3971	(19.7)	38.3	1405	(35.3)	1415	(35.6)	0.2
Japan Coma Scale^a^										
Grade 0 (alert)	913	(22.1)	4453	(26.0)	-11.1	912	(22.9)	925	(23.2)	0.2
Grade 1 (delirium)	925	(22.4)	5443	(31.7)	-21.5	924	(23.2)	946	(23.8)	0.7
Grade 2 (sommolence)	422	(10.2)	1788	(10.4)	0.7	420	(10.6)	398	(10.0)	2.6
Grade 3 (coma)	901	(21.8)	4185	(24.4)	-3.4	899	(22.6)	887	(22.3)	-2.6
missing	967	(23.4)	1289	(7.5)	43.9	825	(20.7)	824	(20.7)	0.0
Preexisting medical conditions										
Neurological diseases	373	(9.0)	2367	(13.8)	-18.6	371	(9.3)	343	(8.6)	0.3
Cardiovascular diseases	892	(21.6)	3676	(21.4)	-6.4	858	(21.6)	853	(21.4)	1.2
Respiratory disease	180	(4.4)	589	(3.4)	3.6	170	(4.3)	165	(4.1)	0.5
Digestive diseases	274	(6.6)	1231	(7.2)	-5.0	268	(6.7)	261	(6.6)	2.9
Metabolic disease	407	(9.9)	1771	(10.3)	-5.2	393	(9.9)	386	(9.7)	0.7
Others	327	(7.9)	1116	(6.5)	0.7	301	(7.6)	271	(6.8)	0.0
Mechanism of injury										
Automobile crash	815	(19.7)	2085	(12.2)	23.8	752	(18.9)	756	(19.0)	0.0
Motorcycle crash	618	(15.0)	2613	(15.2)	3.2	608	(15.3)	631	(15.9)	-1.4
Bicycle crash	270	(6.5)	2129	(12.4)	-15.9	270	(6.8)	271	(6.8)	1.2
Pedestrian traffic accident	294	(7.1)	1559	(9.1)	-3.9	289	(7.3)	294	(7.4)	0.0
Other vehicle	44	(1.1)	122	(0.7)	4.5	41	(1.0)	40	(1.0)	1.0
Fall from high place or Stairs	1418	(34.4)	6366	(37.1)	1.5	1391	(34.9)	1366	(34.3)	-0.4
Machine injury	61	(1.5)	98	(0.6)	9.3	50	(1.3)	47	(1.2)	-0.9
Falling object or Explosion	100	(2.4)	283	(1.6)	6.7	98	(2.5)	89	(2.2)	0.0
Compression injury	175	(4.2)	368	(2.1)	13.1	155	(3.9)	165	(4.1)	1.1
Train	16	(0.4)	163	(0.9)	-5.2	16	(0.4)	15	(0.4)	0.0
Sports	96	(2.3)	182	(1.1)	10.5	91	(2.3)	91	(2.3)	0.7
Other blunt injury	90	(2.2)	322	(1.9)	3.7	88	(2.2)	98	(2.5)	-0.7
Penetrating	62	(1.5)	336	(2.0)	-2.4	62	(1.6)	55	(1.4)	0.0
missing	69	(1.7)	532	(3.1)	-7.8	69	(1.7)	62	(1.6)	0.0
Injury distribution with AIS ≥ 3										
Head	1963	(47.6)	9357	(54.5)	-18.9	1916	(48.1)	1902	(47.8)	-1.0
Face	84	(2.0)	263	(1.5)	3.9	81	(2.0)	76	(1.9)	0.0
Neck	24	(0.6)	115	(0.7)	-1.4	24	(0.6)	21	(0.5)	1.4
Chest	2294	(55.6)	8183	(47.7)	22.8	2197	(55.2)	2153	(54.1)	0.0
Abdomen	552	(13.4)	1686	(9.8)	13.7	521	(13.1)	512	(12.9)	-0.3
Spine	916	(22.2)	2969	(17.3)	11.9	862	(22.5)	862	(21.7)	1.2
Extremity	1208	(29.3)	4270	(24.9)	13.7	1144	(28.7)	1092	(27.4)	0.7
Skin	8	(0.2)	26	(0.2)	3.3	7	(0.2)	8	(0.2)	0.6
Injury Severity Score										
16 to 24	1792	(43.4)	8685	(50.6)	-19.7	1750	(44.0)	1817	(45.7)	-4.4
25 to 34	1421	(34.4)	5700	(33.2)	5.3	1370	(34.4)	1359	(34.1)	2.9
≥ 35	915	(22.2)	2773	(16.2)	19.2	860	(21.6)	804	(20.2)	2.0
Accident day										
Working day	2876	(69.7)	12343	(71.9)	-5.3	2780	(69.8)	2745	(69.0)	1.9
Weekend	1252	(30.3)	4815	(28.1)	5.3	1200	(30.2)	1235	(31.0)	-1.9

^a^ recorded pre-hospital vital signs

Total may not become 100% due to rounding off

*HEMS* helicopter emergency medical services, *GEMS* ground emergency medical services, *SD* standardized difference, *AIS* abbreviated injury scale

**Fig. 2:**
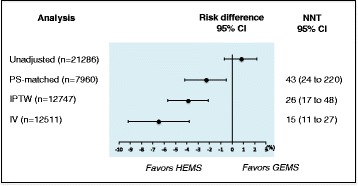
Risk difference in the in-hospital mortality between HEMS and GEMS. PS, propensity score; IPTW, inverse probability of treatment weighting; IV, instrumental variable; HEMS, helicopter emergency medical services; GEMS, ground emergency medical services; CI, confidence interval; NNT, number needed to treat

There were no significant differences in in-hospital mortality between patients transported by HEMS and GEMS in unmatched patients (22.4% [*n* = 924] vs. 23.2% [*n* = 3973]; risk difference 0.8% [95% CI −0.7 to 2.2]); however, significant differences were observed in the propensity score-matched patients (22.2% [*n* = 882] vs. 24.5%, [*n* = 974]; risk difference −2.3% [95% CI −4.2 to −0.5]; number needed to treat 43 [95% CI 24 to 220]) (Fig. 2). In the propensity-score IPTW and IV analyses, we identified 12,747 eligible patients (HEMS 2629; GEMS 10,118). Significant differences were observed (20.8 vs. 23.9%; risk difference −3.9% [95% CI −5.7 to −2.1]; number needed to treat 26 [95% CI 17 to 48]) in the IPTW analysis (Fig. 2).

In the IV analysis, the null hypothesis that there was no association between pattern of HEMS call and actual HEMS use was rejected, with *P* < .001, and an F statistic of 2763. As noted in the Additional file 1, we observed improved balance in covariates across the categories of our instrument compared with the pooled sample. There were significant differences in the in-hospital mortality between HEMS and GEMS (risk difference −6.5% [95% CI −9.2 to −3.8]; number needed to treat 15 [95% CI 11 to 27]) (Fig. 2).

Table 2 shows subgroup analyses of in-hospital mortality between propensity-matched groups. HEMS was associated with lower mortality than GEMS among patients injured by falls, compression-type injuries, and chest and extremity (including pelvic) injuries (AIS ≥3).

**Table 2:** Subgroup analyses of in-hospital mortality rates between propensity score-matched groups

	Matched groups				
	HEMS, %	No. of deaths/Total No.	GEMS, %	No. of deaths/Total No.	*p Value*
Mechanism of injury					
Automobile crash	18.0	(135/752)	17.5	(132/756)	.80
Motorcycle crash	19.1	(116/608)	19.7	(124/631)	.80
Bicycle crash	30.7	(83/270)	29.2	(79/271)	.69
Pedestrian traffic accident	42.9	(124/289)	41.8	(123/294)	.79
Other vehicle	41.5	(17/41)	25.0	(10/40)	.12
Fall from high place or Stairs	21.1	(293/1391)	26.6	(364/1366)	.001
Machine injury	22.0	(11/50)	23.4	(11/47)	.87
Falling object or Explosion	14.3	(14/98)	19.1	(17/89)	.38
Compression injury	16.1	(25/155)	31.5	(52/165)	.001
Train	31.3	(5/16)	46.7	(7/15)	.38
Sports	8.8	(8/91)	2.2	(2/91)	.05
Other blunt injury	25.0	(22/88)	22.4	(22/98)	.68
Penetrating	19.4	(12/62)	29.1	(16/55)	.22
missing	24.6	(17/69)	24.2	(15/62)	.95
Injury distribution with AIS ≥3					
Head	32.6	(625/1916)	32.0	(609/1902)	.69
Face	22.2	(18/81)	34.2	(26/76)	.10
Neck	20.8	(5/24)	33.3	(7/21)	.34
Chest	23.4	(514/2197)	29.1	(627/2153)	<.001
Abdomen	31.3	(163/521)	31.6	(162/512)	.90
Spine	12.9	(111/862)	14.1	(126/894)	.46
Extremity	23.7	(271/1144)	33.9	(370/1092)	<.001
Skin	14.3	(1/7)	37.5	(3/8)	.31
Hospital type					
Tertiary	22.2	(877/3951)	24.7	(937/3798)	.01
Secondary	17.2	(5/29)	20.3	(37/182)	.70

*HEMS* helicopter emergency medical services, *GEMS* ground emergency medical services, *AIS* abbreviated injury scale

Table 3 shows initial vital signs at the emergency department in the unmatched and propensity-matched groups. In comparison with HEMS, the proportion of zero vital signs patients (systolic blood pressure, heart rate, and respiratory rate) was greater and the proportion of normal respiratory rate patients was smaller in the propensity-matched GEMS group than those in the unmatched group. In addition, when we excluded those patients in traumatic arrest on arrival to the emergency department from our propensity score-matched groups, there were no significant differences between HEMS (*n* = 3727) and GEMS (*n* = 3606) for in-hospital mortality (17.4% [*n* = 648] vs. 16.9% [*n* = 610], *p* = .59).

**Table 3:** Initial vital signs at emergency department in the unmatched and propensity score-matched groups

	Unmatched groups					Matched groups				
	HEMS		GEMS		SD, %	HEMS		GEMS		SD, %
	*n* = 4128	(%)	*n* = 17158	(%)		*n* = 3980	(%)	*n* = 3980	(%)	
Systolic blood pressure, mm Hg										
0	272	(6.6)	1342	(7.8)	-4.6	253	(6.4)	374	(9.4)	-11.1
1 to 89	522	(12.6)	1900	(9.4)	10.2	496	(12.5)	455	(11.4)	3.4
90 to 109	552	(13.4)	2157	(10.7)	8.3	537	(13.5)	507	(12.7)	2.4
≥ 110	2725	(66.0)	14506	(71.9)	-12.8	2642	(66.4)	2579	(64.8)	3.4
missing	57	(1.4)	234	(1.2)	1.8	52	(1.3)	65	(1.6)	-2.5
Heart rate, beats/min										
0	272	(6.6)	1342	(7.8)	-4.6	253	(6.4)	374	(9.4)	-11.1
1 to 59	263	(6.4)	1063	(6.2)	0.8	255	(6.4)	279	(7.0)	-2.4
60 to 99	2461	(59.6)	10350	(60.3)	-1.4	2384	(59.9)	2320	(58.3)	3.3
≥ 100	1065	(25.8)	3990	(23.3)	5.8	1024	(25.7)	892	(22.4)	7.7
missing	67	(1.6)	413	(2.4)	-5.7	64	(1.6)	115	(2.9)	-8.8
Respiratory rate, breaths/min										
0	272	(6.6)	1342	(7.8)	-4.6	253	(6.4)	374	(9.4)	-11.1
1 to 9, > 29	719	(17.4)	2708	(15.8)	4.3	689	(17.3)	697	(17.5)	-0.5
10 to 29	2881	(69.8)	11725	(68.3)	3.2	2787	(70.0)	2554	(64.2)	12.4
missing	256	(6.2)	1383	(8.1)	-7.4	251	(6.3)	355	(8.9)	-9.8
Japan Coma Scale										
Grade 0 (alert)	1033	(25.0)	4524	(26.4)	-3.2	1012	(25.4)	1072	(26.9)	-3.4
Grade 1 (delirium)	806	(19.5)	3784	(22.1)	-6.4	775	(19.5)	775	(19.5)	0.0
Grade 2 (sommolence)	510	(12.4)	1920	(11.2)	3.7	489	(12.3)	418	(10.5)	5.7
Grade 3 (coma)	1022	(24.8)	3559	(20.7)	9.8	992	(24.9)	840	(21.1)	9.0
missing	757	(18.3)	3371	(19.6)	-3.3	712	(17.9)	875	(22.0)	-10.3
Glasgow Coma Scale										
≧ 14	2176	(52.7)	9283	(54.1)	-2.8	2101	(52.8)	2169	(54.5)	-3.4
9 to 13	797	(19.3)	3419	(19.9)	-1.5	765	(19.2)	742	(18.6)	1.5
≦ 8	1088	(26.4)	3771	(22.0)	10.3	1049	(26.4)	884	(22.2)	9.8
missing	67	(1.6)	685	(4.0)	-14.6	65	(1.6)	185	(4.6)	-17.4
Body temperature, °C										
< 35	344	(8.3)	1273	(7.4)	3.3	328	(8.2)	278	(7.0)	4.5
≧ 35	2845	(68.9)	12985	(75.7)	-15.2	2769	(69.6)	2917	(73.3)	-8.2
missing	939	(22.7)	2900	(16.9)	14.6	883	(22.2)	785	(19.7)	6.1

Total may not become 100% due to rounding off

*HEMS* helicopter emergency medical services, *GEMS* ground emergency medical services, *SD* standardized difference

Although time from fire department dispatch to emergency department arrival was longer for HEMS than GEMS (median 60 min vs. 35 min), time from emergency department arrival to blood transfusion was shorter for HEMS than GEMS (median 26 min vs. 36 min) in the propensity-matched groups (Table 4).

**Table 4:** Time outcomes between propensity score-matched groups

	Matched groups				
	HEMS	(IQR)	GEMS	(IQR)	*P Value*
Dispatch to ED arrival, minutes, median*	60.0	(48-74)	35.0	(27-46)	<.001
ED to blood transfusion, minutes ≤60, median**	26.0	(12-44)	36.0	(20-50)	<.001
ED to definitive care, minutes ≤90, median***	58.0	(42-74)	60.0	(34-75)	.73

Definitive care indicates surgery or transarterial embolization

*HEMS; *n* = 3746, GEMS; *n* = 3720

**HEMS; *n* = 335, GEMS; *n* = 195

***HEMS; *n* = 278, GEMS; *n* = 263

*HEMS* helicopter emergency medical services, *GEMS* ground emergency medical service, *ED* emergency department, *IQR* interquartile range

## Discussion

In this study, we performed propensity score and IV analyses of 21,286 severely injured patients transported by HEMS or GEMS to 192 hospitals throughout Japan using data from a nationwide trauma database. There was a significant difference between HEMS and GEMS in the mortality rate of adult patients with ISS ≥16 after major trauma. There was also a significant difference between in-hospital mortality for patients transported by HEMS and GEMS after falls from a height, compression injuries, severe chest injuries, extremity (including pelvic) injuries and traumatic arrest on arrival to the emergency department. HEMS assistance thus resulted in an average of 2.3 to 6.5 lives saved per 100 HEMS dispatches for severely injured patients.

In previous studies, the numbers of lives saved per 100 HEMS dispatches have been reported to range from 1.1 to 19 [41–43]. These figures were comparable to those from our study. However, it may be difficult to compare our results directly with those in previous studies because of the large variations in study design, geographical setting, organization of trauma systems, type of pre-hospital trauma care, study population, and definition of mortality.

Our results showed no significant difference in mortality following head injury between the HEMS and GEMS groups. It may be that mortality following head injury cannot be reduced even though HEMS provides pre-hospital neuro-intensive treatments. Such treatments may improve functional outcomes among survivors of head injury. However, our database did not include functional outcomes or post-discharge outcomes.

Random assignment of patients to HEMS or GEMS may be impossible for ethical reasons. Previously published international studies have thus been based on observational studies [3]. The current study provides stricter analyses of HEMS and GEMS than previous studies. Propensity score matching and IPTW analyses can balance covariates that can cause an imbalance between treated and control groups. IPTW analysis can also estimate average treatment effects [31, 32]. For observational and nonrandomized studies, propensity scores represent one of the best available methods to adjust for baseline differences. In addition, to overcome bias from unmeasured confounding factors, we additionally performed an IV analysis. Vital signs are affected by many factors, and ISSs do not account for degree of emergency, such as the existence of airway obstruction caused by persistent hemorrhage from maxillofacial trauma. We thus cannot recognize the presence of airway obstruction, which often needs emergency intervention at the pre-hospital settings, based only on a patient’s vital signs and ISS. In this situation, airway obstruction may become unobserved confounder. IV analysis can theoretically adjust for these unmeasured confounders.

Several previous studies used propensity score matching analysis to compare mortality between HEMS and GEMS in major adult trauma [6–11]. However, it remains unclear which aspect of helicopter transport is responsible for the mortality benefit. HEMS crews with pre-hospital airway management skills have been suggested as one of the possible explanation for any reduction in trauma mortality seen in HEMS-transported patients. Our results indicate that HEMS is associated with higher rates of respiratory function recovery and reduced mortality in patients with chest compression injuries (chest AIS ≥ 3). HEMS was also associated with reduced rates of traumatic arrest on arrival to the emergency department. These findings suggest that HEMS may have a favorable effect on respiratory dysfunction in the pre-hospital setting, and may reduce the number of dead-on-arrival cases.

A helicopter is a means of transportation, not a method of treatment. Any benefit noted with HEMS transport must logically be related to a decrease in the time from injury to definitive care or stabilizing treatment. However, in this study, the median time from dispatch to emergency department arrival was longer in the HEMS than in the GEMS group. In addition, there were no significant differences between HEMS and GEMS in time from emergency department arrival to definitive care, such as emergency surgery or transarterial embolization. Only the time to blood transfusion after emergency department arrival was shorter in the HEMS group. The main reason why blood transfusions may have occurred earlier is that HEMS physicians are able to order the use of universal donor or uncrossmatched blood products during pre-hospital care, permitting transfusions immediately after the patient arrives at the emergency department. These results may indicate HEMS’ pre-hospital care and early blood transfusion can confer favorable conditions before definitive treatment, and these conditions may affect favorable outcomes. Indeed, a previous study suggested that early blood transfusion at the pre-hospital stage was associated with a significant reduction in mortality [44].

When we excluded patients who were dead on arrival from the matched groups, the favorable mortality outcomes for HEMS were lost. The measured variables of this group were well balanced. In this study, we excluded patients who were in traumatic arrest at the time GEMS arrived on the scene. Even if a patient goes into traumatic arrest after GEMS assessment, activated HEMS often reaches the patient and is able to perform resuscitative procedures such as thoracotomy with aortic clamping prior to transporting the patient to the tertiary care hospital. We therefore believe traumatic arrest patients may be less likely to be transported by GEMS after HEMS is alerted. These results thus suggest that the effectiveness of HEMS may lie in pre-hospital intervention rather than in-hospital treatment.

### Limitations

The present study has several limitations. First, information on pre-hospital interventions or HEMS crews was unavailable. Previous studies have argued that a prolonged pre-hospital time might be caused by additional on-scene treatment [5]. The potential survival benefit from HEMS has been suggested to depend on rescue teams possessing superior experience in managing trauma patients resulting in extended preclinical procedures and a benefit on survival. However, our data contained no information on the pre-hospital procedures of HEMS. Thus, role of skilled physicians and nurses remained uncertain. Second, we excluded patients with moderate or minimal conditions (ISS <16) and who were transferred from other hospitals. Thus, the results cannot be generalized to less severely injured patients or transferred patients. Third, to handle missing data, we categorized the continuous values of vital signs and included a category for missing values. However, the categorization of continuous data generally results in a loss of information. Fourth, although a propensity score method was used to adjust for differences in baseline characteristics and injury severity, bias could still be present in the form of confounders that were not measured. IV analysis can theoretically adjust for such unmeasured confounders.

## Conclusion

This nationwide registry-based study identified the benefits of HEMS for patients with serious but potentially survivable injuries. We observed a substantially reduced mortality rate in adult patients with major trauma transported by HEMS compared with GEMS after adjusting for measured and unmeasured confounders.

## Additional file

Additional file 1: Patient Characteristics and Covariate Balance of Pooled Sample and Instrumental Variable Analysis. Table that illustrates patient characteristics and covariate balance relative to fire department HEMS usage for pooled sample and instrumental variable analysis. (XLSX 16 kb)

## Abbreviations

AIS: Abbreviated injury scale
CI: Confidence intervals
GEMS: Ground emergency medical services
HEMS: Helicopter emergency medical services
IPTW: Inverse probability of treatment weighting
ISS: Injury severity score
IV: Instrumental variable.
JTDB: Japan Trauma Data Bank
